# Developments in Neurovascular Diseases and Treatments

**DOI:** 10.1155/2015/608607

**Published:** 2015-08-31

**Authors:** Robert M. Starke, Stephen J. Monteith, Andrew M. Southerland, R. Webster Crowley, Nohra Chalouhi, Dale Ding, David M. Hasan, Aaron S. Dumont

**Affiliations:** ^1^Department of Neurological Surgery, University of Virginia, Charlottesville, VA, USA; ^2^Department of Neurosurgery, Swedish Neuroscience Institute, Seattle, WA, USA; ^3^Department of Neurology, University of Virginia, Charlottesville, VA, USA; ^4^Department of Neurological Surgery, Thomas Jefferson University, Philadelphia, PA, USA; ^5^Department of Neurological Surgery, University of Iowa, Iowa City, IA, USA; ^6^Department of Neurological Surgery, Tulane University, New Orleans, LA, USA

Vascular diseases of the cerebral circulation represent a complex and diverse spectrum of pathology. Within the cerebrovascular field, there have been numerous recent advances in patient diagnosis, evaluation, imaging analysis, medical therapies, microsurgical treatments, and minimally invasive therapeutic modalities. These improvements have been driven by research developments from* in vitro*,* in vivo*, translational, and clinical studies. We review and add to the primary literature concerning this special issue.

Stroke is a major cause of morbidity and mortality. Although intravenous tissue plasminogen activator has been a validated medical therapy in patients with acute ischemic stroke, there are many patients who fail to meet the defined time window for treatment. Neuroprotective agents for acute stroke could provide a major therapeutic advantage for many patients. In this issue, H.-K. Liew et al. demonstrate that “Granulocyte-Colony Stimulating Factor Increases Cerebral Blood Flow via a NO Surge Mediated by Akt/eNOS Pathway to Reduce Ischemic Injury.” Further studies are indicated to better define potential alternative medical therapies in acute ischemic stroke patients. Although earlier randomized clinical trials (RCTs) challenged the use of endovascular therapy in acute cerebrovascular occlusion [1–3], refinements in patient selection and three recent RCTs have validated the benefit of endovascular mechanical thrombectomy, preferably with retrievable stents (i.e., stentrievers), in patients with large vessel occlusion [4, 5]. Limitations of prior RCTs of endovascular stroke intervention included the use of earlier generation thrombectomy devices. In this issue, O. Ozdemir et al. provide us with “Predictors of a Good Outcome after Endovascular Stroke Treatment with Stent Retrievers”.

Long-term follow-up in the International Study of Unruptured Intracranial Aneurysms (ISUIA) [6] and the International Subarachnoid Hemorrhage Aneurysm Trial (ISAT) has validated both microsurgical clipping and endovascular coiling in the treatment of ruptured and unruptured aneurysms [7]. As these outcomes are based on patients who were primarily treated during the 1990s, they may be based on antiquated means of patient evaluation and treatment. Recently, there have been many advances in medical therapies, imaging, microsurgical treatment, and endovascular therapies for cerebral aneurysms [8]. In the current issue, N. Ajiboye et al. provide us with an update on “Unruptured Cerebral Aneurysms: Evaluation and Management.” Cerebral vasospasm continues to be a leading cause of morbidity and mortality following aneurysm rupture. Recent RCTs have not provided us with further therapeutic strategies [9]. Perhaps refinements in patient evaluation and selection will improve patient outcomes. S. H. Sheen et al. provide a protocol on “Intravenous Flat-Detector Computed Tomography Angiography for Symptomatic Cerebral Vasospasm following Aneurysmal Subarachnoid Hemorrhage.” These new imaging modalities may yield a less invasive means for the evaluation and treatment of patients with aneurysmal subarachnoid hemorrhage.

Recent RCTs and prospective cohort studies have challenged current management strategies for unruptured cerebral arteriovenous malformations (AVMs), but these analyses have not been without limitations [10, 11]. When indicated, current AVM interventions include microsurgery, radiosurgery, and embolization. These therapeutic options are also often used individually or as part of a multimodality approach. In this issue N. Ajiboye et al. provide us with an update through “Cerebral Arteriovenous Malformations: Evaluation and Management.” Additionally, S. Peschillo et al. provide us with a review on treatment options in “Brain AVMs: An Endovascular, Surgical, and Radiosurgical Update.”

Neurovascular diseases are a heterogeneous group of disorders that are often associated with poor clinical outcomes. S. Tabuchi provides us with a review on “Auditory Dysfunction in Patients with Cerebrovascular Disease” and A. Y. Lu et al. provide us with an assessment of “Hemifacial Spasm and Neurovascular Compression.” Although the majority of neurovascular diseases are primarily arterial pathologies, cerebral venous diseases may also incur significant morbidity. To round out this issue, we provide an update and review of “Endovascular Treatment of Venous Sinus Stenosis in Idiopathic Intracranial Hypertension: Complications, Neurological Outcomes, and Radiographic Results.” Although cerebrospinal fluid diversion has been a mainstay of treatment in patients with idiopathic intracranial hypertension (IIH) refractory to medical therapy, recent advances in endovascular therapies have provided an effective alternative treatment option. Specifically, venous sinus stenting has been shown to provide durable neurological improvement in IIH patients with venous sinus stenosis and a significant physiologic pressure gradient. Further studies regarding associated complications, neurological outcomes, and radiographic results are necessary.

Improvements in clinical evaluation, imaging, and treatment will lead to better outcomes in patients with neurovascular disease. Although there continue to be many advances in neurovascular disease, these options must be carefully assessed and compared to existing modalities to ascertain the best practices for our patients. Within this compilation of studies, we hope to elucidate areas of uncertainty, recent developments, and clinical necessity.

*Robert M. Starke* *Robert M. Starke* *Stephen J. Monteith* *Stephen J. Monteith* *Andrew M. Southerland* *Andrew M. Southerland* *R. Webster Crowley* *R. Webster Crowley* *Nohra Chalouhi* *Nohra Chalouhi* *Dale Ding* *Dale Ding* *David M. Hasan* *David M. Hasan* *Aaron S. Dumont* *Aaron S. Dumont*
